# Multiple Sclerosis: Inflammatory and Neuroglial Aspects

**DOI:** 10.3390/cimb45020094

**Published:** 2023-02-08

**Authors:** Giulio Papiri, Giordano D’Andreamatteo, Gabriella Cacchiò, Sonila Alia, Mauro Silvestrini, Cristina Paci, Simona Luzzi, Arianna Vignini

**Affiliations:** 1 Neurology Unit, Ospedale Provinciale “Madonna del Soccorso”, 63074 San Benedetto del Tronto, Italy; 2 Section of Biochemistry, Biology and Physics, Department of Clinical Sciences, Università Politecnica delle Marche, 60100 Ancona, Italy; 3 Neurology Unit, Department of Experimental and Clinical Medicine, Università Politecnica delle Marche, 60100 Ancona, Italy

**Keywords:** multiple sclerosis, mitochondrial dysfunction, neurodegeneration, autoimmunity

## Abstract

Multiple sclerosis (MS) represents the most common acquired demyelinating disorder of the central nervous system (CNS). Its pathogenesis, in parallel with the well-established role of mechanisms pertaining to autoimmunity, involves several key functions of immune, glial and nerve cells. The disease’s natural history is complex, heterogeneous and may evolve over a relapsing-remitting (RRMS) or progressive (PPMS/SPMS) course. Acute inflammation, driven by infiltration of peripheral cells in the CNS, is thought to be the most relevant process during the earliest phases and in RRMS, while disruption in glial and neural cells of pathways pertaining to energy metabolism, survival cascades, synaptic and ionic homeostasis are thought to be mostly relevant in long-standing disease, such as in progressive forms. In this complex scenario, many mechanisms originally thought to be distinctive of neurodegenerative disorders are being increasingly recognized as crucial from the beginning of the disease. The present review aims at highlighting mechanisms in common between MS, autoimmune diseases and biology of neurodegenerative disorders. In fact, there is an unmet need to explore new targets that might be involved as master regulators of autoimmunity, inflammation and survival of nerve cells.

## 1. Epidemiology, Etiology, Onset, Disease Course

Multiple sclerosis (MS) is a chronic demyelinating disorder of the central nervous system (CNS) characterized at its core by inflammation involving the gray and white matter of the CNS in a multifocal pattern. It results in demyelinating lesions, focal areas of inflammation characterized by myelin sheath damage surrounded by leukocyte infiltration (macrophages, mast cells, lymphocytes), blood–brain barrier (BBB) breakdown, but also complement and immunoglobulin deposition [1]. Within these areas, inflammation sustained by entry of peripheral cells coexists with damage to neural and synaptic elements while reactive glial elements are engaged in variable terms in cell debris clearance, myelin sheath repair and restoration of neuroaxonal functions [2]. MS is the most common among acquired demyelinating disorders and therefore is considered the most characteristic and prototypical. Symptomatic onset mostly occurs in the age range 20–40, although onset at younger or older ages is not infrequent [3]. The disease is most common in Caucasian populations dwelling in northern latitudes, while exhibiting a lower prevalence in populations dwelling in Africa and in Eastern Asia [4]. It has been observed that people migrating to countries with a lower prevalence appear to have some reduction in the risk of developing the disease [5].

Due to age distribution and prevalence, which is estimated to be as high as 100/100,000 in Western countries, it is regarded as the most frequent cause of non-traumatic disability among young people, affecting women more frequently than men [4].

Etiology of the disease is unknown and highly debated. Currently, the disease’s mechanisms express themselves via a complex interaction between genetic susceptibility, hormonal factors, environmental stimuli and the neuroimmune axis, resulting in CNS directed autoimmunity. 

As for environmental factors contributing to disease pathogenesis, a risk coming from low ultraviolet light exposure and low blood vitamin D has been suggested by the association with a higher prevalence in northern countries and reduced incidence in people migrating during adolescence from northern latitudes to warmer climate areas [6,7,8]. Other environmental factors thought to confer a greater susceptibility to develop the disease are smoking and obesity, hypothetically through their influence on inflammation and immune functions [9,10]. Among other lifestyle factors, it has been recently proposed that sleep deprivation at younger ages might increase the risk of developing MS later in life [11].

It is not yet clear whether sleep disorders might precede the disease; nonetheless, prolonged sleep deprivation has been found in experimental models to be mechanistically related to proinflammatory signaling axes within the CNS, such as microglial phagocytic activation, and to impact synaptic maintenance and myelination [12,13].

At variance from other autoimmune diseases, a single antigen either able to kickstart the disease process in humans or to transfer it to a recipient organism has not been defined, although the presence of a sustained antibody response against intracellular antigens is well established. In this regard oligoclonal bands, which are a cornerstone of MS diagnosis, despite being detectable in other diseases, are thought to derive mainly from production of autoantibodies against ubiquitous intracellular components [14]. 

Several pathogens have been proposed as triggers for disease onset, especially viruses from the *Herpesviridae* family, such as *Epstein–Barr virus* and *human herpes virus 6* [15,16,17,18]. In addition, viral DNA as well as antibodies, directed against viral antigens, have been isolated more frequently in plasma and cerebrospinal fluid (CSF) of subjects with the disease with respect to controls. However, evidence of causal association so far has been inconclusive [17,19]. Other viruses considered to have a relationship with the disease onset are *human endogenous retroviruses* (HERV), whose activation as transposable elements of the human genome might influence disease progression [20,21]. 

Several antigens might contemporarily contribute to disease onset or exacerbation. In this regard, infection from intracellular bacteria such as *Chlamydia pneumoniae* has been associated with MS onset [22], while contact with bacterial superantigens, such as toxins from *Staphylococcus aureus*, has been associated with disease onset and/or exacerbation [23].

Infections from other bacteria, such as *Spirochetes*, *Campylobacter*, *Mycoplasma*, *Chlamydia*, *Bartonella*, *Mycobacteria and Streptococcus,* have been linked to MS development, although to date these pathogens have not been directly isolated from CSF of patients [24]. Helminthic infections, on the other hand, have been reported as potentially protective against MS development [23]. Inflammation, once triggered, might progress through an asymptomatic phase where demyelinating lesions appear in a multiphasic, asynchronous pattern in non-contiguous sites, sometimes asymptomatically, thus configuring the phenomena of dissemination in time and space [25].

The prototypical clinical onset is constituted by acute neurological dysfunction, developing over hours or days, sustained by inflammation of discrete areas of the CNS, such as optic nerves as well as cerebral, brainstem or spinal sensorimotor pathways. Dysfunction coming from disease attacks usually resolves in a partial or complete manner. According to the current consensus definition, a single disease episode suggestive of MS, but not sufficient to fulfill criteria for dissemination in time and space, is termed clinically isolated syndrome (CIS) [26]. On the other hand, a condition where lesions suggestive of a demyelinating disease are present in the absence of clinical manifestations is termed radiological isolated syndrome (RIS) [27]. Over time, according to differences in lesion appearance, clinical manifestations and disability build-up, the disease might assume a relapsing-remitting (RRMS) or progressive course, which could be further distinguished between primarily (PPMS) or secondarily progressive (SPMS). While the former is characterized by repetition of brief episodes of neurologic dysfunction sustained by acute CNS inflammation and synchronous appearance of demyelinating lesions, in the latter, neurologic functioning, especially in motor systems, slowly decays over time regardless of the appearance of new demyelinating plaques. Progressive courses can be further distinguished into secondary or primary according to whether they have been preceded by a longstanding RRMS or not [28,29]. The occurrence of relapses and sustained progression of the disease are not mutually exclusive. Less frequently, a benign disease course, characterized by absence of relapses with conservation of neurologic functioning over decades, even without immunomodulatory therapy, has been described [30]. On the other hand, very aggressive courses, with high lesional loads, such as tumefactive MS [31] or a monophasic fulminant onset ab initio, have also been described [32]. 

## 2. Pathology: Demyelination

On a histologic basis, typical MS lesions consist of confluent foci of inflammatory myelin breakdown, centered on perivascular spaces close to cerebral venules and surrounded by reactive gliosis, which affect both white and gray matter of the brain and spinal cord. Aspects of parenchymal damage are combined with a varying degree of infiltration of blood-borne cells, such as CD4^+^, CD8^+^ lymphocytes and monocytes, entering through focal areas of BBB disruption. In addition, surrounding astroglial, microglial and oligodendroglial cells display a reactive phenotype [33,34]. 

Lesion types have been further subdivided into different patterns according to the major constituents of inflammatory infiltrates and CSF characteristics, potentially underlying nuances in their pathophysiology. Pattern I lesions display T cell and macrophage infiltration, while pattern II lesions show in addition antibody and complement deposition, suggesting a contribution of humoral mechanisms to disease pathology. Pattern III is characterized by distal oligodendrogliopathy with dysregulated myelin protein expression and oligodendrocyte apoptosis, which still occurs on an inflammatory background. A fourth pattern, which has been described in rarer cases, is characterized by oligodendrocyte degeneration occurring in the white matter surrounding plaques [35,36]. 

Lesions in the gray matter show more pronounced alterations in structure and numbers of synapses than their white matter counterparts [37]. Perivenular spaces, i.e., perivascular spaces surrounding venules, are thought to be a critical area of immune cell trafficking from peripheral organs, and demyelinating lesions are thought to originate from confluence of foci of inflammation surrounding these spaces [38].

Lesional activity has been characterized according to the relative preponderance of inflammation, tissue destruction and gliosis/repair processes. Active lesions are distinguished by increased permeability of the BBB and a significant infiltration of dendritic cells, B, CD4^+^, CD8^+^ T lymphocytes, mast cells, monocytes from the periphery, cytokine and adhesion molecule expression, coexisting with activated microglia. On the other hand, inactive lesions are characterized by a minor inflammatory component at their core, relatively preserved integrity of the BBB and presence of sparse phagocytes and microglia at the lesion border. Both active and inactive lesions exhibit neuroaxonal loss, whereas inactive ones might expand slowly over time [38]. Despite greater BBB integrity in chronic lesions, magnetic resonance imaging (MRI) studies in MS patients have detected impaired glymphatic flow, which appears to be more prominent in advanced disease [39]. 

In RRMS and PP/SPMS, both types of lesions coexist, albeit in different proportions. In fact, inactive lesions are thought to be the most common type of lesion in both forms, although active ones are more common in RRMS, underlying a direct pathophysiologic impact of acute inflammation. Progressive forms of the disease, on the other hand, show inactive slowly expanding lesions, while displaying aggregates of inflammatory cells resembling tertiary lymphoid follicles in leptomeningeal compartments combined with global CNS atrophy. Relative proportions of B, plasma cells and T cells also vary [33,40].

Both active and inactive lesions show histologic signs of impaired axonal transport, such as anterograde and retrograde axonal degeneration. These changes, albeit to a lesser degree, also occur in apparently normal gray and white matter in parallel with meningeal inflammation, microglial activation, gliosis and synaptic loss [41]. 

In addition to inflammation and axonal degeneration, other important features of MS pathology on a cellular level are alterations in synaptic morphology and numbers, iron deposition and mitochondrial changes. Iron deposition might take place in apparently normal white matter, in lesions, but also in basal nuclei [42]. 

Iron deposition begins in the earliest phases of the disease, increasing with age and is thought to contribute to oxidative stress and disability progression [43,44]. Mitochondria in MS are altered in numbers and distribution, displaying a reduced expression of components of the oxidative phosphorylation chain [45,46,47]. 

Functional aspects of mitochondrial impairment will be further discussed in the following sections given their critical relationship with neuroaxonal loss. Another important histologic feature of MS is the loss of glial cells and neurons, which might be operated by heterogeneous pathways [48]. Observations from autoptic studies and animal models suggest that mechanisms of cell death might express themselves through a continuous spectrum encompassing apoptosis, ferroptosis and also necroptosis [48,49,50]. 

Further enquiry is needed to elucidate details about the relevance of distinct mechanisms of cell death in MS over its natural history. The histopathological picture of MS also comprises remyelination, characterized by the formation of thin myelin sheaths around damaged axons, either sustained by activation of oligodendrocyte progenitor cells or by terminally differentiated oligodendrocytes [51,52]. These processes will be briefly described in the following sections.

## 3. Remyelination

Remyelination is a process which may be distinguished into repair of damaged myelin or de novo synthesis. It is thought to be operated in the CNS either by activation of terminally differentiated oligodendrocytes or by recruitment and migration of staminal precursors known as oligodendrocyte progenitor cells (OPCs) [53,54]. OPCs are also thought to be critical for tuning inflammation and angiogenesis; furthermore, they possess complex electrophysiological properties and are thought to form synapses with neurons [55,56].

It is well accepted that remyelination constitutes a continuous and ubiquitous process occurring within the CNS, although it is unclear whether it might be sufficient in restoring myelin function in lesioned areas. Some lesions, in fact, undergo an incomplete repair, characterized by formation of thin sheaths surrounding axons, especially at the lesion border [57]. These areas are defined as “shadow plaques” and are thought to be areas where the remyelination process has come to a halt [58]. It is not well known whether they are the result of single or repeated demyelinating processes, whether they are more prone to subsequent remyelination or whether they might harbor a quiescent recovery potential, but intriguingly, these areas are devoid of OPC elements and, therefore, myelin restoration is thought to be operated only by mature oligodendrocytes [59]. 

Remyelination declines with aging; it is regulated by synaptic activity but is also highly influenced by the secretory and signaling activity of astroglial elements, as well as by iron transport and phagocytic activity of macrophages and microglial cells [60,61]. Chronic inflammation might impair remyelination dynamics, yielding incomplete repair of damaged sheaths. In accordance with this hypothesis, it has been reported that in addition to shadow plaques, slowly expanding lesions, characteristic of progressive disease, possess a lower remyelination potential [62].

It is currently under debate whether in humans de novo myelination might be more effective in repairing injured structures in comparison to activation of differentiated oligodendrocytes. It has, however, been esteemed that only 0.3% of oligodendroglial elements are regenerated per year; therefore, activation of differentiated elements appears of crucial importance, as well as the mechanisms that might render this process more efficient [59]. Remyelination has also been shown to reverse the alteration in mitochondrial numbers observed in demyelinated axons, suggesting a potential in counteracting neuroaxonal loss [63]. On the whole, remyelination appears as a fundamental process in MS with the potential to preserve functioning of sensory and motor systems and, therefore, delaying and limiting disability.

Several biochemical cascades, involving lipid metabolism, cholesterol efflux, retinoid-X-receptor α dependent pathways, phagocytosis, but also epigenetic regulation through histone deacetylases, have been implied as potential mechanistic targets [64,65,66,67]. Among these, leucine-rich repeat and Ig domain-containing 1 (LINGO-1), a glycoprotein expressed by neurons, and OPCs, whose blockade has been shown to improve myelination in animal models of the disease, has been proposed as a promising target for remyelination [68]. Opicinumab, a monoclonal antibody targeting LINGO-1, has failed in trials to reach its efficacy endpoints, despite showing at the highest doses and in younger patients a small improvement in disability worthy of further research [69].

## 4. Pathogenesis: Immunologic Perspectives

### 4.1. Mechanisms Pertaining to T and B Lymphocytes

Pathogenesis has been mostly studied through animal models either involving immunization against CNS antigens, infection with neurotropic viruses or administration of neurotoxic/myelinotoxic compounds, such as lysolecithin or cuprizone [70].

The most commonly adopted models derive from parenteral administration of myelin-derived peptides, such as myelin basic protein (MBP), myelin oligodendrocytic glycoprotein (MOG) and proteolipid protein (PLP) complexed with adjuvants, which results in an inflammatory demyelinating disease of the CNS, termed experimental allergic encephalomyelitis (EAE). These models have allowed researchers to characterize in detail some pathogenetic aspects useful to extrapolate data for therapy development, although they do not allow reproduction of every aspect of the human disease, especially concerning its multiphasic clinical course [71]. Other animal models involve infection with neurotropic viruses, such as Theiler’s encephalomyelitis virus (TMEV) [72].

Evidence from genomic studies suggests a critical role of loci involved in antigen presentation, such as HLA DRB1*15:0, in conferring susceptibility to the disease, while other HLA haplotypes, such as the A*02 and B*44, have been associated with a protective effect [5]. 

HLA genes can be distinguished into three classes: class I and class II HLA encode for major histocompatibility complex (MHC) proteins, which are crucial for antigen presentation, while class III HLA loci encode for molecules involved in the inflammatory cascade, such as complement proteins, tumor necrosis factors (TNFs), 21-hydroxylase and heat shock proteins [73,74].

MHC class I molecules (encoded by HLA-A, HLA-B and HLA-C loci), present intracellular self- or non-self-antigens to CD8^+^ cytotoxic T cell receptors and killer cell immunoglobulin-like receptors (KIR) [75]. On the other hand, class II molecules (encoded by HLA-DP, HLA-DQ and HLA-DR) are expressed on the membrane of antigen-presenting cells (such as macrophages, B cells and dendritic cells) and serve the function of displaying short antigen peptides to CD4^+^ helper T cells [76]. 

In addition to HLA loci, more than 200 non-MHC-coding genomic variants have been reported to confer susceptibility to MS, albeit with different effect sizes. In fact, many of these variants affect genes involved in immune system pathways, such as interleukin 2 receptor subunit α (IL-2RA), but also intronic and intragenic sequences related to splicing and quantitative gene expression [77]. Daclizumab, which inhibits IL2RA, has shown high clinical efficacy in preventing MS relapses, although it has been withdrawn for hyperacute hepatotoxicity [78].

As for effector mechanisms, in accordance with autoptic data, pathogenesis shows great similarities to T-cell-mediated diseases; therefore, a central role has been theorized for CD4^+^ and CD8^+^ lymphocytes [79,80]. The former, when primed towards their proinflammatory T_h1_ and T_h17_ phenotypes, are thought to be important directors of the immune response towards the CNS [38], while the latter, primed to their cytotoxic phenotypes, are the predominant cell type, under a quantitative perspective, surrounding demyelinated axons [33,81,82].

Many currently approved therapies for MS modulate various aspects of T cell function, including response to activating stimuli, functional polarization, egress from lymph nodes, migration and CNS entrance [83]. Glatiramer acetate is thought to modulate T helper cell polarization toward a Th_2_ phenotype, dampening CNS-directed inflammation [84].

In addition, recent studies on distinct immune system cell subtypes in MS highlight the role of several regulatory subpopulations of the innate and adaptive immune system in balancing disease severity, such as forkhead box protein 3 (FOXP3) positive CD4^+^ cells, Tr1-positive CD4 cells [5], CD56^bright^ NK cells [85]. Subsets of anti-inflammatory CD8^+^ cells have been described, but to date a single surface antigen combination conferring this functional phenotype has not been defined [85,86,87]. 

In recent decades, evidence from animal and clinical studies supported an important role for B cells, given their role in tuning T cell function, antigen presentation, autoantibody production and also in leptomeningeal lymphoid follicle formation [40]. A similar role is shared with dendritic cells, which orchestrate T cell activation through similar processes [88]. B cells comprise a heterogeneous host of naïve, memory and effector subpopulations also including tolerogenic and anti-inflammatory subsets, collectively termed as B_regs_, characterized by production of IL-10, IL-35 and TGF-β [89].

In clinical studies, B cell-directed anti-CD20 antibodies (especially ocrelizumab) have shown significant benefits and have been approved in both RRMS and PPMS, where they possess lesser efficacy [90]. Their long-lived therapeutic effects might derive from their ability to blunt proliferation of proinflammatory clones such as mature naïve B cells and memory B cells with a parallel stimulation of regulatory populations, such as IL-10-producing B cells, including autoreactive regulatory clones [5,91]. Another very important target for B cell physiology, closely related to MS, is constituted by Bruton tyrosine kinase (BTK), a master regulator of B-cell activation, whose expression is not exclusive to B cells since it has also been detected in myeloid T cells and osteoclasts. It is considered as a “rheostat” of proinflammatory signaling and a regulator of autoreactive cells [92]. Besides tuning B cell receptor (BCR) activation, BTK takes part in signaling of toll-like and Fc receptors, modulating the inflammatory response; therefore, its excessive activity has been related to autoimmunity [93,94]. In addition to its effects on immune activation and inflammation cascades, a recent in vivo study on cultured cerebellar slices interestingly shows that BTK activity is upregulated after lysophosphatidylcholine and metronidazole-induced demyelination, while its inhibition might hasten myelin repair, suggesting complex effects on the CNS [93].

The observed effectiveness, in both animal models and human subjects, of BTK inhibitors in several autoimmune diseases further strengthens the hypothesis that regulation of this cascade might be of therapeutic value [95]. Currently, two brain-penetrant BTK inhibitors, evobrutinib and tolebrutnib, are being assessed with regard to their effectiveness in preventing MS relapses and in reducing disease activity [94].

Several studies have observed a shift in energy metabolism affecting lymphocytes, macrophages and dendritic cells towards aerobic glycolysis (the so-called Warburg effect) in association with dysfunctional oxidative phosphorylation in T lymphocytes [96,97]. It is not known whether this process, termed “metabolic reprogramming”, might be the cause or a consequence of aberrant immune activation.

### 4.2. Mechanisms Pertaining to Innate Immunity

Given the predominance of immune-mediated mechanisms in animal models and the clinical responses associated with immune modulators, several efforts have been made to elucidate the relationship between specific cytokines and disease phenotypes, but also between effector mechanisms of the innate immune system, such as the kinin or complement cascade, and MS pathogenesis. Among cytokines, Th_1_ and Th_17_ cytokines are considered pivotal proinflammatory signals, while IL-10, IL-27, IL-35 and especially type I interferons have been associated with disease amelioration [5,98]. 

TNF-α blockade has been shown to trigger significant disease exacerbation [99,100], while immunomodulation via interferon β (IFN-β) has shown significant clinical efficacy in preventing disease relapses [101]. IFN-β is thought to target antigen presentation processes, modulate cytokine secretion, T cell polarization and MHC molecule expression, although recent studies have also suggested that the activity of the cGAS-STING pathway, a critical regulator of endogenous type I IFN production, might constitute an important determinant of the effectiveness of interferon therapy [98,102,103]. 

The cGAS-STING pathway, in brief, is considered an intracellular “damage-sensing” cascade involved in innate immunity that is primarily activated by binding of cGAS (cyclic GMP-AMP synthase) to exogenous/endogenous double-stranded DNA fragments outside of the cell nucleus [104]. It has been suggested that its activity might be altered during infections but also in several brain inflammatory disorders [98]. cGAS activation produces 2′5’-cyclic adenosine monophosphate guanosine monophosphate (2′5′-cGAMP), which activates tank-binding kinase 1 (TBK1) and IκB kinase (IKK), inducing STING (stimulator of interferon genes) oligomerization [105,106,107]. STING activation leads to phosphorylation and activation of interferon regulatory factor 3 (IRF3) and nuclear factor kappa-light-chain-enhancer of activated B cells (NF-κB), which upregulate the type I interferon response, upregulating expression of interferon regulated genes, in turn regulating the synthesis of TNF-α, IL-1β and IL-6, but also of the STING protein [106]. It has been recently observed that RRMS patients might exhibit in peripheral blood mononuclear cells a downregulated activity of the cGAS-STING/IFN-β-axis, while also displaying a reduced expression of interferon regulated genes [103]. It has been therefore suggested that interferon therapy might be mostly effective in patients with a downregulated endogenous response, perhaps in addition to pharmacological modulation of STING activity [105].

Among other soluble signals of innate immunity, studies in EAE models have highlighted a role for bradykinin (BK) in modulating cytokine secretion and CNS lesion development [108]. BK type 1 receptor (B1) activation is thought to mediate BBB breakdown and increased vascular permeability, favoring inflammation [109]. In EAE models, enalapril administration has been shown to increase plasma BK concentration and reduce clinical and pathological severity, while B1 receptor blockade counteracted the protective effects of enalapril [110]. In human studies, increased B1 receptor expression has been detected in T lymphocytes isolated from peripheral blood of MS patients with respect to control subjects, suggesting a potential role in CNS inflammation [111]. As for the complement system, autoptic studies have shown involvement in myelin phagocytosis within acute lesions, but also persistent deposition in chronic lesions in PPMS as well as in gray matter lesions [112]. The complement system might play a complex role in disease pathogenesis since its effects are not limited to debris clearance, but also to processes related to survival cascades. Evidence from cell models has shown that astrocytes secrete complement proteins when stimulated by proinflammatory stimuli as TNF α, IL-1β and IL-8 [113], while sublytic levels of C5b-C9 proteins might drive antiapoptotic responses in oligodendroglial elements [114]. The complement system is also thought to play an important role in removal and maintenance of synaptic structures [115,116]. Evidence from plasma and CSF biomarker studies shows a trend towards increased concentration of complement components, such as C1q, C3 and C4 in RRMS, SPMS and PPMS, as well as an increase in endogenous inhibitors, such as factor H, suggesting heightened complement activity in all forms of the disease [117,118].

These observations might therefore constitute a rationale for assessing complement regulation as a therapeutic target; in a small series of patients, eculizumab, a C5 inhibitor, has recently shown a discrete tolerability in a small series of MS patients, with no severe adverse drug reactions nor disease relapses, supporting further clinical assessment [119].

Data from experimental models suggest that no single antigen or effector cell type might be sufficient to summarize every pathogenetic aspect of MS, whose immunopathogenesis is multifactorial and underlies an interaction, in the periphery and in the CNS, between proinflammatory stimuli, specific subtypes of immune cells and host-specific factors. Among host-specific factors, polygenic susceptibility, hormonal influences, epigenomic regulation, gut microbiome signals and environmental factors (including pollutants and smoking) might shape disease activity [120]. 

It has been proposed that the gut microbiome may alter the MS immunopathological framework at least by a dual mechanism. In fact, gut dysbiosis, i.e., imbalance between tolerogenic and proinflammatory commensals, might promote inflammation in remote sites, while molecular mimicry between gut antigens and CNS epitopes might select autoreactive cell clones. Gut microbes produce metabolites that also directly target the CNS. It was observed that dietary tryptophan may be metabolized through the serotonin, indole and kynurenine pathways into components that act as aryl hydrocarbon receptor agonists [121] and exert anti-inflammatory actions mediated by astrocytes [122]. The effectiveness, in preclinical models, of the immunomodulatory drug laquinimod, which however failed to reach significant clinical endpoints [123], is thought to derive from its effect on glial aryl hydrocarbon receptors [124]. In addition, dimethylfumarate, a drug approved to treat RRMS, has been found to reduce bacterial production of neurotoxic phenol and indole catabolites of phenylalanine and tryptophan [125].

Under this perspective, the efficacy of the so-called “immune reconstituting” therapies, such as cladribine, alemtuzumab and bone marrow transplantation, might depend not only on quenching of acute inflammation, but also on reconfiguring the immune repertoire to a point that allows previously suppressed cells to emerge and affect immunologic processing in distant sites [126,127]. Ocrelizumab, despite requiring maintenance therapy, could be considered, due to its long-lived effects, as a drug with a profile of action closely comparable to immune reconstituting therapies [128]. 

On the whole, acute inflammation, which is preponderant in RRMS, is considered highly dependent on the entrance in the CNS of pathogenic autoreactive cells from the periphery and might lead over time to formation of persistent meningeal tertiary lymphoid structures [129]. In accordance with this hypothesis, it has been observed that therapies targeting infiltration of autoreactive cells in the CNS, such as natalizumab, an anti-very late antigen 4 (VLA4) monoclonal antibody, are far more effective in RRMS [130,131], while they might induce devastating disease rebounds after a prolonged discontinuation [132].

In addition, another recently discovered layer of regulation of inflammatory activity is represented by endogenous transposable elements, such as *human endogenous retroviruses*, whose activation has been linked to both disease relapses and progression [21,23]. Recently, temelimab, a monoclonal antibody directed against the HERV-W envelope protein, has shown discrete tolerability in small MS cohorts and promising effects on radiological markers [133].

Despite the abundance of data highlighting the predominance of mechanisms pertaining to the innate and adaptive immune system in sustaining acute attacks, several signaling pathways pertaining to astrocytic, oligodendrocytic and microglial elements appear of primary importance in poising disease activity and determining neuron survival from the earliest phases [134].

## 5. Pathogenesis: A CNS-Centered Perspective

### 5.1. Role of Glial Cells

In MS, in analogy with other neurologic diseases, the degree of neurologic dysfunction and disability relates to the extension of damage to several functionally distinct circuits, which are composed of high-order networks of neuronal and glial cells. Glial cells are specialized elements that sustain neurons through several processes. For instance, astrocytes tune the surrounding microenvironment including pH, water and ion content according to neuronal metabolic demands, but also scavenge free radicals and participate directly in synaptic transmission [135]. Microglia are mesenchymal-derived immunocompetent cells whose principal functions are considered clearing cell debris through phagocytosis and coordinating inflammatory responses within the CNS [136], whereas oligodendrocytes, which reside exclusively in the CNS, are neuronal lineage cells specialized in myelin synthesis, maintenance and repair [137]. 

**Figure 1:**
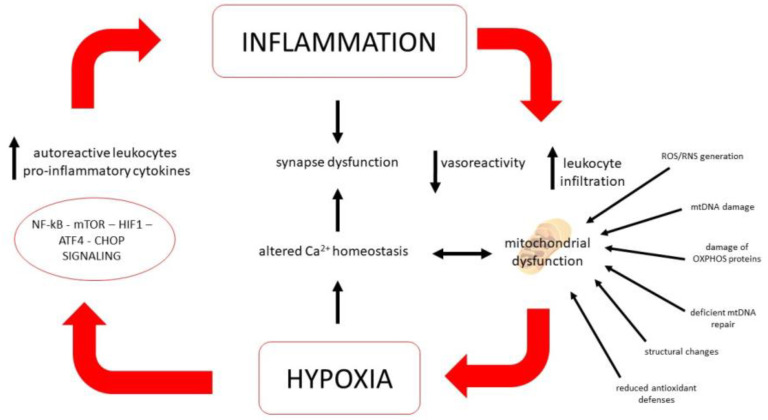
The positive feedback loop of hypoxia and inflammation. The low oxygen presence will lead to the activation of NF-κB, m-TOR, HIF1, ATF4, CHOP signaling, all regulators of inflammation. Increased levels of autoreactive leukocytes and pro-inflammatory cytokines can decrease vasoreactivity, and impair mitochondrial function, which could in turn exacerbate hypoxia.

On the whole, glial cells are highly plastic and specialized elements that might alter their phenotype, integrating various electrical and molecular stimuli, including those produced by an inflammatory environment. Their signals directly tune the immune response within the CNS and vascular/BBB permeability, but also functional properties of neural cells. They secrete chemokines and cytokines, might change their morphology according to the surrounding microenvironment and their signals pose a significant influence on neuronal survival and functioning of the tripartite synapse [138]. Several efforts have been made to define functionally distinct subsets of astrocytes or microglial cells with either a neuroprotective or neurotoxic phenotype, although the heterogeneity of activation states and phenotypes of glial cells suggests the existence of a continuous spectrum, rather than distinct subcategories [139]. Therefore, the interplay between the immune system, glial cells and neurons might shape disease progression precociously, triggering processes that might follow a divergent direction from acute inflammation [34]. Hence, acute and chronic inflammation constitute stressors that might recruit, in the long run, signaling pathways tied to responses to neurotoxic insults such as protein misfolding, loss of membrane integrity and nucleic acid damage, yielding profound biochemical changes on a cellular level that ultimately impact on ion homeostasis (especially calcium and iron), growth-factor signaling, remyelination and cell survival cascades [140]. Such processes are all mechanistically tied to mitochondrial oxidative phosphorylation, calcium buffering and redox balance and, therefore, are considered common effectors of cell loss in MS and neurodegenerative diseases (Figure 1). During MS’s earliest phases they are considered to be mainly triggered by autoimmunity, but whether their activation might progress independently from inflammation is currently under debate [46].

### 5.2. Role of Mitochondria

Considering the pathophysiological analogies found in different experimental models of neurodegeneration, indicating a significant role of mitochondria in regulating cell fate, several lines of research have converged on mitochondrial impairment and related mechanisms in shaping MS pathology [45,48]. In fact, under a pathogenetic perspective, it has been observed in several disease models that the high energy consumption of nerve cells and their reliance on oxidative metabolism might render them particularly vulnerable to degenerative changes in contexts of impaired mitochondrial ATP production, potentially starting multiple interlinked deleterious processes. 

It is well documented that mitochondria from MS patients are altered in morphology and distribution, carrying mutations in mtDNA while also showing diminished expression of elements of the respiratory chain and altered expression of heat shock proteins, resulting in ATP production impairment. Such changes are thought to be more relevant in progressive disease, albeit beginning from the earliest phases [46,47].

In accordance with the hypothesis of a pivotal role of mitochondria in MS, many cytotoxic agents, used to induce demyelination in animal models, such as cuprizone, lysolecithin or ethidium-bromide, may directly alter their number or disrupt respiratory chain complex expression [70,141,142]. 

Decreased energy production might alter ionic transmembrane gradients, sustaining calcium entry, heightening reactive oxygen species (ROS) generation, endoplasmic reticulum stress and stimulation of intracellular transducers, such as activating transcription factor 4 (ATF4), glucose regulated protein (GRP78) and C/EBP homologous protein (CHOP), which are closely tied to apoptosis and inflammation [143,144,145]. These interlinked processes might concur to decrease cell energy metabolism progressively, promoting a self-sustaining cycle of damage, which might lead to cell death, with consequent debris release and inflammation. 

Such a sequence of events has been termed a “mitochondrial spiral” and is thought to occur in Alzheimer’s dementia and stroke [146,147]. Despite striking differences in clinical course between stroke, primarily neurodegenerative diseases and MS, dysfunctional energetic homeostasis appears as a shared pathogenetic factor of critical importance [148].

### 5.3. Ion Homeostasis and Energy Metabolism Regulation

In MS, reduction in cerebral blood flow might impair ATP production, especially within demyelinated areas, whereas altered numbers and morphology of mitochondria might reflect a homeostatic response to increased metabolic demands or relative lack of oxygen and nutrients. Under a pathophysiological perspective, oxygen–glucose deprivation, leading to decreased ATP production, promotes sodium accumulation and calcium entry from the extracellular space, operated by the sodium calcium exchanger (NCX) reverse mode [149,150]. In an analogy to the biochemical changes triggered by hyperacute oxygen–glucose deprivation happening during ischemia, significant accumulation of sodium ions within active and inactive demyelinated areas has been detected through MRI in MS patients [151], supporting the hypothesis of lasting imbalances in calcium cycling. 

Although NCX has been mostly perceived to promote excessive calcium influx after hypoxia, its reverse mode transport (sodium-dependent calcium influx) might be essential for the activation of ischemic conditioning [152] and might also play a pivotal role, due its close physical and functional coupling with neuronal and glial sodium-dependent glutamate transporters, in sustaining glutamate-induced ATP synthesis [152,153,154]. Further enquiry about the functional properties of distinct isoforms that display heterogeneous gating properties [155] and differential distribution within the CNS [156] is needed in order to clarify their pathophysiological significance.

In an analogy with data from acute damage models, a potential role for NCX in chronic neurodegenerative diseases, which is to be further characterized, has been suggested [157]. In parallel, sodium accumulation within demyelinated axons might modify the activity of other sodium-dependent transporters, including the sodium hydrogen exchanger (NHE) and sodium potassium chloride cotransporter (NKCC). These transporters have been implied in regulating cell death across several pathologic scenarios in the CNS, although the precise functional interactions between sodium-dependent transporters, energy metabolism, substrate uptake, inflammation and calcium cycling in MS need to be further elucidated. 

Ultimately, deregulation of calcium homeostasis, among its widespread toxic effects, contributes to oxidative phosphorylation and ATP synthesis impairment, affecting at first detrimentally those functions mostly dependent on sustained energy synthesis, such as synaptic transmission and plasticity [158]. In addition, prolonged calcium accumulation, either by entrance through the plasma membrane or by excessive loading of internal stores, triggers mitochondrial-dependent pathways, such as MPTP opening, resulting in irreversible mitochondrial membrane depolarization and thus cell death [159]. 

In parallel to mechanisms inducing mitochondrial dysfunction during hypoxia, several signaling pathways related to cellular resistance to such challenges have been explored for a potential role in mitigating neuroaxonal loss in MS. In particular, signaling cascades related to ischemic conditioning, autophagy and metabolic reprogramming have stood out in preclinical models, also with potential implications for aberrant immunologic processing (Figure 1) [160]. 

At present, experimental models have highlighted a significant integration between the activation of cytoprotective pathways involved in preserving mitochondrial function during hypoxia, which affect oxidative stress and calcium overload, and the master signaling pathways modulating cell energy metabolism and inflammatory signaling [161].

The activity of potassium-dependent ATP channels, which are among the effectors of ischemic conditioning, has been associated with amelioration of the disease in preclinical models [162]. 

A common effector of cell responses to hypoxia and a key player in ischemic conditioning is hypoxia-inducible factor 1α (HIF1) [163]. Reduced energy production in MS might trigger HIF1 activation, adapting CNS cell energy metabolism, angiogenesis and ROS production to context-dependent cues, but also influencing iron accumulation and apoptosis [164]. On the other hand, sustained inflammation might heighten HIF1 activation and ROS production in continuously stimulated lymphocytes, leading to impaired responsiveness and senescence [165]. HIF1 induces expression of several genes, including vascular endothelial growth factor B (VEGF-B), whose levels have been found to be lower in MS patients during stable disease with respect to control subjects, suggesting that disease activity and metabolic stress might be associated [166]. 

A potential relationship between cascades involved in hypoxia and MS might be supported not only by evidence from experimental models, but also by the observation that remote ischemic preconditioning, a procedure thought to stimulate cell programs aimed at preserving the integrity of mitochondrial functions, might improve gait dysfunction in progressive MS patients [167,168,169].

Among regulators of metabolic programming, a critical cell pathway, implied in tuning the shift of metabolic resources in accordance to nutrient availability, is represented by mechanistic targeting of rapamycin (mTOR), a ubiquitous regulator of energy metabolism, proliferation and inflammation [170,171]. 

In addition to its well-known effects on lymphocyte proliferation and suppression, its role in diverting utilization of metabolic resources in glia and nerve cells by repurposing their phenotype towards anabolism or catabolism is being increasingly recognized, especially for potential implications for autophagy and remyelination [172]. 

Indeed, it has been observed that mTOR plays an important role in shifting metabolism towards aerobic glycolysis in activated microglia [96]. In addition, preliminary evidence coming from human studies has also suggested a potential efficacy in MS of therapies acting on the mTOR axis, either indirectly, such as metformin, or directly, such as rapamycin [173]. Among other cascades functionally related to the mTOR axis, recent studies have suggested involvement of DJ-1 and parkin, whose functional roles are tied to oxidative stress and mitophagy, in driving inflammation as well as cell death in MS [174,175,176].

### 5.4. Oxidative and Cell Stress Signaling Pathways

From a wider perspective, among other regulators of cellular resistance to metabolic stressors, klotho, an anti-aging protein involved in the FGF-23 signaling, oxidative stress and mitochondrial damage [177] has been recently associated with remyelination in experimental models and has also been suggested as a potential player in MS pathogenesis. In accordance with this hypothesis, it has been found in higher titers in serum from MS patients with respect to controls [178,179,180]. 

The involvement of cellular pathways related to aging is also suggested by the observation that sirtuins (SIRT), ubiquitary NAD-dependent enzymes which are also critical for lifespan regulation and epigenetic regulation, might influence disease phenotype in EAE. More specifically, it has been suggested that SIRT might contribute to MS pathogenesis by regulating oxidative stress, mitochondrial phosphorylative oxidation and autophagic networks [181]. In addition, they might poise activation of master regulators of inflammation such as NfKB, modulate antigen presentation by dendritic cells and activate either anti-inflammatory or pro-inflammatory responses. In particular, SIRT1 overexpression has been found to ameliorate EAE phenotype [88]. SIRT might also act in conjunction with nuclear erythroid factor 2 (Nrf2), a transcription factor involved in antioxidant production, mitochondrial biogenesis and oxidative phosphorylation, which is thought to play a role in neurodegeneration and MS pathogenesis [182].

Dimethylfumarate, a drug approved to treat RRMS, exerts a complex action on pathways regulating B and T cell survival, promoting emergence of regulatory cell subsets, while in neurons its effects are tied to Nrf2 activation, with a potential cytoprotective effect [183]. 

Another interesting area of research, which lies at the border between cell survival cascades, metabolism and stress signaling, is constituted by signaling lipids, such as sphingosine and ceramides. Ceramides in particular are considered a crucial switch for apoptosis due to their regulation of mitochondrial outer membrane potential and permeability [184]. Fingolimod and siponimod, oral therapies acting on sphingosine-1-phosphate receptors (S1PRs), have shown clinical efficacy by downregulating their expression on peripheral lymphocytes and thus inhibiting lymphocyte homing towards the CNS [185]. 

Follow-up of patients treated with S1P analogues has highlighted a potential protective effect on brain atrophy in addition to regulating immune cell trafficking [186], which might be related to fine-tuning in both neurons and astrocytes of lipid signaling cascades pertaining to endo/exocytotic vesicle cycling and neurotransmitter release. It has also been observed that these drugs possess the ability to dampen glial inflammatory phenotype changes induced by the disease [187,188,189].

### 5.5. Synaptic Aspects

In parallel with inflammation and the associated dysfunctional energy metabolism in the CNS, synapses exhibit a precocious dysfunction, which over time might become a bona fide “synaptopathy” [37]. Summarizing evidence from pathological studies, significant alterations in synaptic numbers and morphology have been described in lesions and within apparently normal gray and white matter underlying long-standing functional alterations [33]. In preclinical models, perturbation in pre- and post-synaptic protein expression, electrophysiology and synaptic demolition by the complement cascade, are well-recognized elements [37,190,191]. The main players involved in these processes are thought to be dysfunctional astrocytes and microglia primed by inflammatory changes, which might retain long-lasting pathologic phenotypes that outlive resolution of acute inflammation. Several efforts have been made to characterize, in more detail, these alterations, leading to a theory of the establishment of a long-lived imbalance between excitatory and inhibitory transmission, progressing to a complex dysfunction of synaptic potentiation and depression [192,193]. Despite morphological and functional abnormalities having been most extensively studied with regard to glutamatergic and GABAergic systems, other neurotransmitters such as neuropeptides and neurosteroids are being increasingly recognized as involved in synaptic dysfunction in MS [190,194]. 

Other less studied mechanisms, although increasingly recognized in MS pathogenesis, are constituted by BBB permeabilization and vascular and endothelial regulation, which are bidirectionally linked to synaptic activity, inflammation and non-physiological neurovascular coupling.

### 5.6. Vascular Aspects 

Several studies have observed in MS elements of endothelial dysfunction, such as an increase in adhesion molecule (i.e., VCAM-1) expression, which might be related to inflammation-driven permeabilization of the BBB [195]. In addition, an epidemiological association between increased incidence of ischemic stroke and migraine, which are characterized by endothelial dysfunction [196], has been reported in MS patients [197,198]. Furthermore, global brain perfusion in MS patients is frequently decreased, suggesting the presence of a widespread disruption of autoregulation [199].

Cerebral vascular reactivity, measured as flow-mediated dilation (FMD) or response to hypercapnia, evaluated with neurosonologic methods, was found to be impaired in patients with SPMS or PPMS in comparison to RRMS [200,201]. Collaterally, a proinflammatory phenotype of platelets, characterized by increased endothelial adhesion, chronic activation, adhesion to astrocytes and neurons, has been described and is thought to promote lesion activity [202].

On the whole, MS appears to embrace and connect various aspects of neuronal and immune system physiology. Most pathogenetic mechanisms considered so far, either involving acute inflammation or dysfunction of neuronal and glial homeostatic processes, while mostly active within lesions, might affect apparently normal gray and white matter. 

It is not well-known, however, which signals influence apparently normal areas at the beginning of the disease. These observations have suggested a potential pathogenetic role for mediators exerting their effects beyond the borders of the inflammatory milieu within lesions. From this perspective, in addition to cytokines, vasoactive peptides do stand out, given their role in the CNS as “volume transmitters”, i.e., neuropeptides released by neurons from sites not restricted to synapses and therefore able to diffuse beyond synaptic borders [203]. 

### 5.7. Role of Neuropeptides

Neuropeptides are ubiquitous signaling molecules that in the PNS and CNS might be expressed by neurons and co-released with fast neurotransmitters, acting on local scales. Neuropeptides might also travel for longer distances, engaging more distant targets, in a manner akin to hormones [203]. Experimental models have shown that several neuropeptides, including endothelin-1 (ET-1), calcitonin gene-related peptide (CGRP) and vasoactive intestinal peptide (VIP) might be involved in regulation of synaptic potentiation/depression [204,205,206]. Additionally, pleiotropic effects including growth factor-like properties and potent regulatory effects on vascular tone, inflammation and immunity have been described [207,208].

**Figure 2:**
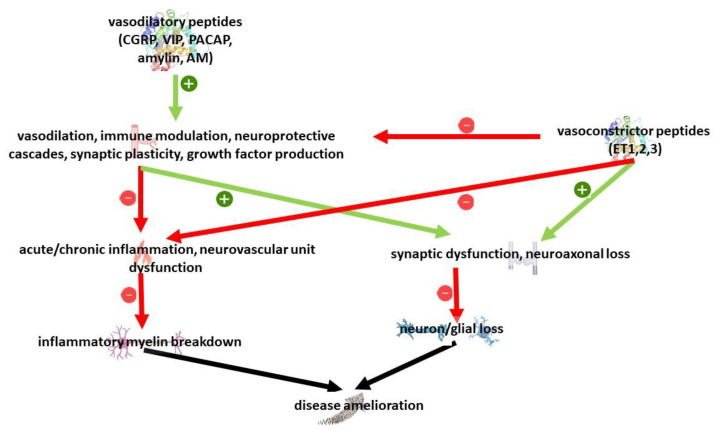
Overview of relevant pathways to MS pathology regulated by vasoactive peptides.

Vasoactive peptides have been mostly studied for their relationship with BBB permeabilization, vascular tone and neurogenic inflammation. Recent research has highlighted in addition pleiotropic neuroprotective but also neurotoxic properties in cell and animal models. On the whole, despite the existence of different isoforms and receptors, vasodilatory peptides have been associated with neuroprotective properties, while vasoconstrictive peptides have been associated with a detrimental effect [209,210]; considering their action on multiple targets, they might be implied in MS pathogenesis at several levels, including innate and acquired immunity, vascular dysfunction but also neuron survival and glial dysfunction (Figure 2).

The vasoactive intestinal peptide (VIP) and pituitary adenylate cyclase-activating peptide (PACAP) have been extensively studied in EAE models, suggesting a complex bidirectional pathogenetic role in inflammation and demyelination. Activation of their shared receptors (VPAC 1-2) has been reported to ameliorate EAE severity, while selective VIP knockout or VPAC-1 receptor knockout or pharmacological blockade has been observed to confer resistance to EAE development [211,212].

Other neuropeptides, such as substance P and neuropeptide Y (NPY), have also been associated to a potential anti-inflammatory role in EAE models. Among vasoconstrictive peptides, the endothelin family (ETs), represented by endothelin 1 (ET-1), endothelin 2 (ET-2) and endothelin 3 (ET-3), stands out for a potential pathogenetic role. ETs are ubiquitous mediators considered potent vasoconstrictors that act on a local scale, with prominent actions on vascular tone, remodeling and endothelial dysfunction [213]. All the three peptides act in synergy on different receptor, resulting in highly regulated signals on vascular tone [214]. Among the three peptides, ET-1, of endothelial origin, is the most studied. On a cellular level ET-1, by activating endothelin A and endothelin B receptors, might modulate neuronal cascades implied in cell survival, such as CHOP and Jun [215], while it is produced by astrocytes following demyelination, with a consequent activation of the notch pathway, which has been associated with defective myelin repair [216,217]. Other investigations have also suggested that ET-1 might exert a pleiotropic role during acute neuronal injury. In fact, a recent study on a spinal cord hypoxia-reperfusion injury model has shown that endothelin receptor blockade might ameliorate tissue damage [218], while He and colleagues have observed that remote ischemic conditioning is abrogated by preemptive ET receptor blockade, thus requiring an increase in ET-1 signaling to stimulate neuroprotective cascades, such as Nrf2 [219]. In addition, ET-1 overexpression has been found to increase disease severity in transgenic mice, while receptor blockade has been associated with diminished EAE progression [220,221]. As for evidence coming from human studies, plasma levels of ET-1 and ET-3 are increased in MS patients with respect to control subjects, and increased CSF ET-1 concentrations have been associated with a poorer visual recovery in MS patients after an episode of optic neuritis [222,223].

VIP, PACAP and CGRP, which are vasodilatory, have been found to reduce the severity of neurologic dysfunction in EAE, via a modulatory action on inflammation and immune activation [224,225,226].

CGRP, as VIP and PACAP, possesses pleiotropic properties, with a contribution from its effects on various cell targets, including vascular smooth muscle, neurons, glia and immune cells [227]. In particular α-CGRP has been proposed as involved in the protective effects of ischemic postconditioning [228]. In murine stroke models, CGRP administration at reperfusion was found to reduce infarct size after middle cerebral artery occlusion [229], while CGRP knockout in a bilateral carotid stenosis model was found to reduce angiogenesis and to increase oxidative damage and demyelination [230]. 

As for inflammation and immune activation, CGRP has been shown to exert a context-dependent bidirectional effect. In particular, it has been found to dampen toll-like receptor (TLR) responses during lipopolysaccharide (LPS) stimulation, but also to poise excessive inflammation during sepsis, hypothetically through cAMP-dependent signaling; furthermore CGRP signaling might influence maturation of CD4^+^- Cd25^+^-FOXP3^+^ lymphocytes [231,232].

In the CNS it is considered a primary effector of neurogenic inflammation in migraine [233], but has also been found to ameliorate EAE severity during disease induction through a complex regulation, in microglial elements, of the expression of proinflammatory immune activation markers, such as IL1-β and IL6, or anti-inflammatory markers, such as Ym1 and CD163 [226]. 

Furthermore α-CGRP is produced in the CNS by spinal motor neurons, which upregulate its synthesis after mechanical injury, such as after axotomy [234], or during inflammation, such as in the acute phase of EAE [235]. CGRP, beyond its role in acute inflammation and immunity, as a neurotransmitter, affects monoaminergic circuits and might be involved in pathophysiology of depression and cognitive impairment, which commonly occur during MS [236,237,238].

Due to its wealth of functions and the ubiquitous expression of CGRP receptors in the CNS [239], it might influence in a pleiotropic manner MS pathogenesis, not only regulating inflammatory cascades, but also through mechanisms involving regulation of growth factor production, survival cascades and synaptic plasticity [227,233,240]. In addition, its potent vasodilatory action might play a role in preserving the integrity of neurovascular unit functioning. Considering data from experimental models of CNS demyelination and the epidemiological association between migraine and MS, a pathogenetic relationship between CGRP and MS pathogenesis appears worthy of further study.

CGRP belongs to the amylin (AMY) family of neuropeptides and is structurally related to adrenomedullin (AM), which might act as a low-affinity agonist on CGRP receptors [241]. Similarly to CGRP, AM was found to reduce EAE severity in experimental models [242] while expression of its mRNA in choroid plexuses was found to be higher in progressive MS patients in comparison to controls in autoptic studies, paralleled by upregulation of other genes involved in neuroprotective cascades, including the HIF axis [243]. 

CGRP also bears a higher structural homology to amylin in comparison to AM [244]. Amylin, closely related to amyloid β (Aβ) and a major constituent of amyloid plaques, has been investigated in several preclinical models of neurodegeneration, which have shown significant effects on neuronal survival and proinflammatory signaling [245,246]. Amylin might, in the first place, exert its effects through binding to AMY receptors, which are involved in modulation cascades relevant to inflammation, energy metabolism and synaptic plasticity. CGRP, on the other hand, due to its structural homology to amylin, displays high affinity towards AMY1a receptors, potentially reinforcing amylin signaling at physiologic levels [231,244,247]. In addition to the effects mediated by signaling through their specific receptors, it has been observed that a hexameric peptide shared by amylin, tau protein, serum amyloid P and Aβ A4 might bind proinflammatory mediators in plasma and reduce polymerization of amyloid fibrils, eliciting a therapeutic effect in EAE [248,249]. Under a speculative perspective, such molecular motifs might also affect neurodegenerative changes, since recent studies have suggested a complex relationship between Aβ metabolism and remyelination [250]. Furthermore, in human biomarker studies, lower CSF Aβ concentration has been associated with a worse prognosis in MS [251]. 

Another potential implication for the amylin family of neuropeptides in MS pathogenesis is supported by the observation that AMY, AM, CGRP and Aβ share common catabolic pathways, represented by endopeptidases such as neprilysin (NEP) [252,253,254,255], endothelin converting enzyme [256] and insulin-degrading enzyme [257,258], which have been described as key players in regulating inflammation and degenerative changes within the CNS [259]. 

At present, NEP appears an interesting target in MS since data coming from experimental studies support a role in ameliorating EAE severity through catabolism of several vasoactive peptides, including those of the amylin family and endogenous opioids such as met-enkephalin [260]. In contrast, data coming from genomic studies point to an epidemiological association between a polymorphism in the MMEL1 gene, encoding NEP2 and MS susceptibility [261], although further studies are needed on this subject. Another enzyme involved in catabolism of vasoactive peptides, which could play a pathogenetic role in MS, is represented by CD26/dipeptidyl peptidase IV (DPP4), which primarily regulates systemic glucose metabolism by catabolizing glucagon-like peptide-1, glucagon inhibiting peptide and glucagon influencing insulin sensitivity and type II diabetes mellitus pathogenesis. More recently, it has also been implied in several brain disorders since its other substrates include neuropeptide Y, secretin, substance PACAP and amyloid peptides [262]. At present, lower soluble DPP4 expression in plasma samples from MS has been detected with respect to controls, while surface expression by CD8^+^ circulating cells was increased [263]. Further studies are needed on the relationship between MS pathogenesis and CD26.

## 6. Conclusive Remarks

In MS, a wealth of mechanisms contemporarily concurs to pathogenesis, crosslinking innate and adaptive immunity, stress response and survival-related cascades in neural and glial cells. Acute and chronic inflammation appear the primary drivers of damage, although neurodegenerative changes, such as synaptic disruption and neuroaxonal loss, display early appearance and might progress independently from the resolution of acute inflammation. Significant clinical progress has been achieved through introduction of highly effective immunomodulating drugs in delaying the onset of disability and clinical conversion in RRMS. Unfortunately, therapeutic tools for progressive forms appear much less effective to date. It is increasingly recognized that disability might progress independently from inflammation, whereas irreversible decay of neurologic function might depend on the exhaustion of neuronal functional plasticity, which compensates for neuroaxonal loss through remyelination, synaptic remodeling and staminal precursor recruitment. In order to extend time to irreversible disability, approaches involving intensive immunosuppression in the earliest clinical phases have been advocated, although it is yet to be ascertained whether such therapies might influence in the long run degenerative processes pertaining to glial and neuronal cells.

Despite the identification of several potential neuroprotective agents in neurodegeneration and EAE models, none have to date shown similar, long-lasting effects in humans. Among current therapy regimens, only dimethylfumarate and S1P receptor agonists, in addition to their immunomodulatory properties acting on high-order regulatory pathways of inflammation, energy metabolism and cell survival, have shown a neuroprotective potential and might therefore speculatively represent a proof-of-concept for further drug development. A current etiology for the disease as well as its precise triggers are not known, although research has shed light in recent years on the existence of several physiological interlinked mechanisms, which might at the same time concur to aberrant immune activation but also to degenerative aspects and whose modulation could constitute an interesting therapeutic target. Among various putative targets, neuropeptides could speculatively play an important role, which is partly sustained by experimental and biomarker studies, in consideration of their ubiquitous distribution and their multifaceted actions on immunity and neuronal processes.

Therefore, in MS, it would be advisable for future studies to identify essential shared cell pathways underlying inflammation, cell proliferation and functional reprogramming of the neuroimmune axis. Such elements could constitute interesting targets for drug design, with potential implications towards other chronic neurologic diseases involving degeneration of nerve cells. In addition, the development of related biomarkers could play a significant role in focusing the field of study.

## Data Availability

Not applicable.
